# Tunneled Fascia Iliaca Catheter Placement for Chronic Pain From Advanced Osteosarcoma

**DOI:** 10.7759/cureus.36475

**Published:** 2023-03-21

**Authors:** William J Kuo, Borna S Shirvani, Kevin Guthmiller, Giustino Varrassi, Omar Viswanath, Sarang Koushik

**Affiliations:** 1 Anesthesiology, Keck School of Medicine of the University of Southern California, Los Angeles, USA; 2 Pain Medicine, Paolo Procacci Foundation, Rome, ITA; 3 Pain Management, Innovative Pain and Wellness, Scottsdale, USA; 4 Anesthesiology, Louisiana State University Health Sciences Center School of Medicine, New Orleans, USA; 5 Anesthesiology, Creighton University School of Medicine, Phoenix, USA; 6 Anesthesiology, Valleywise Health Medical Center, Phoenix, USA

**Keywords:** tunneled catheter, orthopedic tumor, palliative treatment, fascia iliaca compartment block (ficb), regional anesthesiology

## Abstract

The fascia iliaca compartment block (FICB) is a regional anesthetic technique for hip and femoral surgery that blocks the femoral, obturator, and lateral femoral cutaneous nerves. We report the case of a middle-aged female patient who presented with excruciating left lower extremity pain secondary to metastatic left femur osteosarcoma. A FICB with the tunneled catheter was sterilely placed in the operating room as palliative therapy due to the difficulty in pain control, as the patient experienced severe somnolence with high-dose opioid therapy. Conventional techniques such as a femoral nerve block were also precluded due to difficult anatomy secondary to tumor compression. Near-total pain relief was achieved postoperatively and lasted over seven weeks until discharge. This case report demonstrates the unique use of the FICB as a primary pain management technique for the control of chronic lower extremity cancer pain.

## Introduction

The fascia iliaca compartment block (FICB) refers to a regional anesthetic technique employed in hip and femoral surgery that blocks the femoral, obturator, and lateral femoral cutaneous nerves [1]. The FICB was first described by Dalens et al. in 1989 as a reliable block alternative to the traditional “three-in-one” femoral nerve block, with a high degree of sensory blockade of the lumbar plexus nerves supplying the thigh [2]. The FICB has been well-documented as an anesthetic technique in case reports for hip and femoral surgeries [3-5]. It has also been used to control pain due to hip fractures in the geriatric population at risk of medication- or anesthetic-induced delirium [6,7]. The primary aim of this case report is to demonstrate the use of a FICB tunneled catheter as a primary pain management technique for the control of chronic lower extremity cancer pain. While it has been theorized in the literature [8], we were unable to locate an existing case report.

This case report was previously presented as a poster at the American Society of Anesthesiologists (ASA) Annual Meeting on October 23, 2022, in New Orleans, LA.

## Case presentation

A middle-aged female patient, with a past medical history of end-stage renal disease on hemodialysis, presented with stage IV osteosarcoma of the left proximal femur that had previously been treated with two surgical resections, multiple courses of chemotherapy, and palliative radiation therapy. Her oncologic course was complicated by known metastatic lesions to the bilateral lungs, left kidney, and thoracic and sacral spine. Our inpatient pain service was consulted by the oncology team for the patient's excruciating left lower extremity neuropathic pain that was inadequately controlled by a multimodal analgesic medication regimen as prescribed by the palliative care service, which included transdermal fentanyl 100 mcg every three days, oral hydromorphone 4 mg every two hours as needed (PRN) for moderate pain, oral hydromorphone 6 mg every two hours PRN for severe pain, intravenous hydromorphone 0.4 mg every two hours PRN for breakthrough pain, oral gabapentin 100 mg every eight hours, oral acetaminophen 1 g every eight hours, and oral methocarbamol 750 mg every eight hours. The patient requested PRN medications at the maximum frequency as ordered. Escalation of her opioid and gabapentinoid regimen was precluded due to increasing cognitive difficulties and somnolence with higher dosages. Her pain was described as constant, sharp, stabbing, and primarily radiating down the anterior thigh but not past the knee; it worsened with any movement and was partially relieved with rest. This pain limited her ability to ambulate and sleep. Physical examination revealed edema in the thigh and leg, active range of motion of the foot and ankle, and refusal to move the knee or hip joint due to pain. Palpation over the inguinal ligament elicited pain. Pain scores prior to consultation ranged from 0 (immediately after opioid administration) to 10 (with motion), with an average score of approximately 7. Table 1 summarizes the patient's analgesic medication regimen.

**Table 1:** Analgesic medication regimen prior to tunneled catheter placement

Analgesic medication	Route of administration	Dose	Frequency
Acetaminophen	Oral	1000 mg	Q8 hours
Fentanyl	Transdermal	100 mcg	Q3 days
Gabapentin	Oral	100 mg	Q8 hours
Hydromorphone	Oral	4 mg and 6 mg	Q2 hours PRN moderate and severe pain
Hydromorphone	Intravenous	0.4 mg	Q2 hours PRN breakthrough pain
Methocarbamol	Oral	750 mg	Q8 hours

A regional nerve block was planned for her unremitting left lower extremity pain. However, a traditional femoral nerve block could not be done due to significant tissue compression from the large size of the tumor, which was 8.5 cm in maximal diameter. As an alternative, a single-shot ultrasound-guided FICB was performed with 20 mL of 0.5% ropivacaine mixed with 4 mg of dexamethasone. The patient reported significant pain relief and did not require PRN opioids for nearly 40 hours postoperatively. Subjectively, her alertness and mood were noted to improve dramatically. Due to the success of this initial block, the patient was brought to the operating room and placed under deep sedation in preparation for a tunneled FICB catheter. Sterile surgical techniques, including surgical scrub, gown, double glove, and an occlusive full-body drape, were followed. A 15 MHz linear ultrasound probe was used to identify the relevant anatomy; notably, ultrasonic windows were limited secondary to tumor burden and thigh edema. With the transducer oriented in a cephalon-caudad manner at the level of the inguinal ligament, a 17-gauge Tuohy needle was carefully advanced in-plane to the target, which was deep to the confluence of the sartorius muscle and internal oblique muscle, and superficial to the iliacus muscle. The fascia iliaca was dissected off the iliacus muscle with normal saline, and an Arrow regional catheter was advanced with an estimated 10 cm deployed past the needle tip. The catheter was then tunneled some 10 cm down the thigh subcutaneously; puncture sites were sealed with Dermabond, and the catheter was dressed securely. The nerve block was maintained with an On-Q pump infusing 0.25% bupivacaine at a rate of 8 mL per hour without patient-controlled boluses. Our plan was to keep it in place for at least two weeks.

Following placement, the patient reported near-total pain relief with no opioid PRN medications required for over four days, when her On-Q pump ball finally needed replacement. The patient reported subjectively improved sleep quality and participation in physical therapy. No additional clinician boluses of bupivacaine were needed. Her left lower extremity pain continued to be well controlled for the remainder of her hospitalization with On-Q pump rates ranging from 4 to 6 mL per hour of 0.25% bupivacaine. The tunneled FICB catheter ultimately remained in place for a total of 49 days during her inpatient admission without any signs or symptoms of infection. She was ultimately discharged by her oncology primary team with our tunneled catheter in situ to a skilled nursing facility capable of accommodating On-Q pump refills in a comfort-care setting. Unfortunately, the patient was ultimately lost to follow-up on subsequent chart review.

## Discussion

Tunneled catheters utilizing a traditional femoral nerve block approach have been reported in case reports on peripheral orthopedic tumors [9,10]; however, our case demonstrates that a tunneled technique may be utilized for nontraditional peripheral blocks such as the FICB, which was our primary outcome. Although current literature reports rare instances (0-3%) of peripheral nerve catheter infections [11], this complication may have severe, even fatal consequences. Our case also demonstrates that with a strict aseptic technique and placement in a sterile operating room environment, a peripheral nerve block catheter may be maintained for at least seven weeks in an inpatient setting with a minimized risk of infection, which was our secondary outcome.

## Conclusions

We discussed the unique use of a tunneled FICB catheter for the treatment of pain caused by advanced lower extremity osteosarcoma, which precluded a traditional femoral nerve catheter due to compressive tumor effects on the patient's anatomy. This case report demonstrates that a tunneled FICB catheter placed under sterile conditions can provide long-term pain relief for chronic lower extremity cancer pain for at least seven weeks without increased risk of infection.
